# Role of ethylene in responses of plants to nitrogen availability

**DOI:** 10.3389/fpls.2015.00927

**Published:** 2015-10-30

**Authors:** M. I. R. Khan, Alice Trivellini, Mehar Fatma, Asim Masood, Alessandra Francini, Noushina Iqbal, Antonio Ferrante, Nafees A. Khan

**Affiliations:** ^1^Department of Botany, Aligarh Muslim UniversityAligarh, India; ^2^Institute of Life Sciences, Scuola Superiore Sant’AnnaPisa, Italy; ^3^Department of Botany, Jamia Hamdard University New Delhi, India; ^4^Department of Agricultural and Environmental Sciences, Università degli Studi di MilanoMilan, Italy

**Keywords:** ethylene, mineral nutrients, nitrogen availability, N use efficiency, phytohormones

## Abstract

Ethylene is a plant hormone involved in several physiological processes and regulates the plant development during the whole life. Stressful conditions usually activate ethylene biosynthesis and signaling in plants. The availability of nutrients, shortage or excess, influences plant metabolism and ethylene plays an important role in plant adaptation under suboptimal conditions. Among the plant nutrients, the nitrogen (N) is one the most important mineral element required for plant growth and development. The availability of N significantly influences plant metabolism, including ethylene biology. The interaction between ethylene and N affects several physiological processes such as leaf gas exchanges, roots architecture, leaf, fruits, and flowers development. Low plant N use efficiency (NUE) leads to N loss and N deprivation, which affect ethylene biosynthesis and tissues sensitivity, inducing cell damage and ultimately lysis. Plants may respond differently to N availability balancing ethylene production through its signaling network. This review discusses the recent advances in the interaction between N availability and ethylene at whole plant and different organ levels, and explores how N availability induces ethylene biology and plant responses. Exogenously applied ethylene seems to cope the stress conditions and improves plant physiological performance. This can be explained considering the expression of ethylene biosynthesis and signaling genes under different N availability. A greater understanding of the regulation of N by means of ethylene modulation may help to increase NUE and directly influence crop productivity under conditions of limited N availability, leading to positive effects on the environment. Moreover, efforts should be focused on the effect of N deficiency or excess in fruit trees, where ethylene can have detrimental effects especially during postharvest.

## Introduction

The classical plant hormone, ethylene has emerged as a potent molecule to regulate numerous physiological and morphological responses in plants by interacting with other signaling molecules (Iqbal et al., 2012; Khan and Khan, 2014; Fiebig and Dodd, 2015; Khan et al., 2015). Ethylene plays an important regulatory roles in plant responses to mineral nutrients availability, such as nitrogen (N; Iqbal et al., 2015), phosphorous (P; Li et al., 2009), potassium (K; Jung et al., 2009), calcium (Ca; Xu et al., 2010), magnesium (Mg), manganese (Mn; Dorling et al., 2011), copper (Cu; Arteca and Arteca, 2007), zinc (Zn; Khan and Khan, 2014) and controls plant responses under both optimal and stressful conditions (Iqbal et al., 2013). The ethylene biosynthesis and plant responses vary with the availability of mineral nutrients (Iqbal et al., 2013).

Nitrogen is an important nutrient required for plant growth and development as it is a core constituent of a plant’s nucleic acid, proteins, enzymes, and cell wall and pigment system (Krapp, 2015). Plants are frequently exposed to N stressed conditions, excess N due to application of N fertilizers or deficiency. While low N limits the growth of crop plants (Iqbal et al., 2015), the loss of excess N fertilizers contributes to environmental pollution (Gastal and Lemaire, 2002). The availability of N is of agricultural concern because plant metabolism is differently affected by excess, optimal and deficient levels (Iqbal et al., 2015). In maintaining the physiological status of plants under these conditions, the role of ethylene in responding to N status in plants has been identified (Tian et al., 2009; Fiebig and Dodd, 2015; Iqbal et al., 2015). The availability of N concentrations modify the effect of ethylene and plant responses, like other mineral nutrients such as phosphate (Li et al., 2009), sulfate (Zuchi et al., 2009), potassium (Shin and Schachtman, 2004), iron (Romera and Alcantara, 1994). Fiebig and Dodd (2015) have recently reported that N supplementation of 10 mM returned ethylene concentrations in over-irrigated *Solanum lycopersicum* plants to the levels of well-drained plants, leading to an increase in shoot fresh weight that correlated with decreased ethylene levels. This can be explained considering that over-irrigation induces nitrate leakage and subsequently N deficiency. Similarly, N differentially regulates proline and ethylene biosynthesis in order to alleviate salt-induced photosynthetic inhibition in mustard plants (Iqbal et al., 2015). It has been also shown that exogenous ethylene (applied as ethephon, an ethylene releasing compound) increases N assimilation and photosynthesis in *Brassica juncea* plants subjected to different levels of N (Khan et al., 2008; Iqbal et al., 2011). In *B. juncea*, Iqbal et al. (2015) have shown that plants exhibited lesser photosynthesis and growth when treated with 5 mM N than 10 mM N, whereas 20 mM N was inhibitory under no-stress condition. This indicated that these levels were low, sufficient and excess, respectively. The inhibitory effect of excess-N was related to high ethylene production, but under salt stress, as the demand for N increased the excess-S optimized ethylene and led to higher proline production and promoted photosynthesis and growth (Iqbal et al., 2015). Similarly, it has been found that a high (10 mM) concentration of N inhibits the lateral root growth of *Arabidopsis thaliana*, although the number and length of lateral roots of the *etr1-3* and *ein2-1* mutants were less affected than wild-type plants. The leaf longevity in *Agropyron cristatum* was affected by ethylene at different N levels (Ren et al., 2013). Plants under low N conditions accelerate the development and usually show early transition to reproductive stage, reaching earlier to senescence stage. Plants grown to high N availability have longer vegetative stage and delayed senescence. In both cases, ethylene has a pivotal role, since it is also known as senescence hormone.

This review explored the state of the art of the information available on the role of N in modulating ethylene responses in whole plant and different plant organs. The information related to ethylene and N availability has been critically discussed arising due to the contrary results obtained in different works. Moreover, the lack of information has been highlighted indicating where further investigations should be addressed.

## N Availability and Ethylene Biosynthesis and Signaling

The literature has only recently started to explore the nature of the relationships between plant hormones and macronutrient signaling. The following pages describe recent advances in the study of the ethylene signaling pathway in the presence of N perturbation and provide new information based on *in silico* analyses.

The availability of N is one of the main factors limiting plant growth and development. Ammonium (NH_4_^+^) and nitrate (NO_3_^-^) are the predominated inorganic forms of nitrogen taken up from the soil. In particularly, nitrates are the most readily available form of N for root absorption because it is not absorbed by colloids. Nitrate is assimilated by higher plants after being reduced to nitrite and then ammonium as a result of the sequential action of nitrate and nitrite reductases, and the NH_4_^+^ can be subsequently assimilated into glutamate and glutamine via the glutamine synthase (GS)/glutamate oxyglutarate aminotransferase cycle (GOGAT) (Crawford, 1995). These metabolic intermediates act as important signaling molecules or as the major amino donors for the synthesis of other amino acids and N-containing compounds, thus sustaining plant growth and development, and plant responses to biotic and abiotic stresses (Stitt, 1999; Forde and Lea, 2007; Vidal and Gutiérrez, 2008; Mur et al., 2012; Renault et al., 2013). The assimilation of N by plants, or its incorporation in plants, depends on the availability of light and activities of photosynthesis because N can only be incorporated if there are enough carbon (C) skeletons.

It is thought that N acts as a signaling element in plants, but very little is known about how this occurs (Lea and Miflin, 2003) or how N interacts with the ethylene biosynthesis and signaling pathway that is closely associated with complex environmental stresses. Ethylene is essential for regulating plant responses to biotic and abiotic stresses, and plays a key role in regulating growth and senescence (Lin et al., 2009). Ethylene production rapidly increases in plants subjected to wounding, flooding, drought, osmotic shock, senescence, ozone, and pathogen/insect invasion (Wang et al., 2002; van Loon et al., 2006; Di Baccio et al., 2012), and this leads to the activation of cell responses through the ethylene signaling pathway and its interactions with the signaling pathways of other plant hormones (Overmyer et al., 2000; Wang et al., 2002; Trivellini et al., 2014). Ethylene is synthesized by two enzymes encoded by small gene families: 1 aminocyclopropane 1 carboxylic acid (ACC) synthase (ACS) and ACC oxidase (ACO). The reaction is first catalyzed by ACS, which converts *S*-adenosyl-L-methionine (SAM) to ACC, and then ACC oxidase catalyzes the conversion of ACC to ethylene with the release of CO_2_ and cyanide (Wang et al., 2002). ACS is the rate-limiting step in ethylene biosynthesis, and controls the main step in stress-induced ethylene regulation (Tsuchisaka et al., 2009), whereas ACO activity is constitutively present in most vegetative tissues. The ethylene biosynthetic pathway is relatively simple, but its production is strictly regulated at various levels. In addition to transcriptional regulation (Tsuchisaka and Theologis, 2004a,b), post-translational regulation is pivotal for developmental and stress-induced ethylene production (Christians et al., 2009; Han et al., 2010; Skottke et al., 2011; Lyzenga et al., 2012).

In order to investigate the role of ethylene depending on N availability, we first listed the genes involved in ethylene biosynthesis, signaling and responses by searching The *Arabidopsis* Information Resource (TAIR^1^) (**Supplementary Table S1**) and then analyzed the publicly available microarray data on the Affymetrix ATH1 microarray platform (as of June 2015) using Genevestigator (Hruz et al., 2008). A similarity search subsequently enabled the determination of lists of the same genes regulated upon a given N perturbation (**Supplementary Table S1**). The analysis considered the expression profiles of the genes that showed a >2-fold change in transcription level (*P* < 0.01) under conditions of nitrate starvation and low or high N content, and the fold-change values were hierarchically clustered by genes and experiments using Euclidean distances.

**FIGURE 1:**
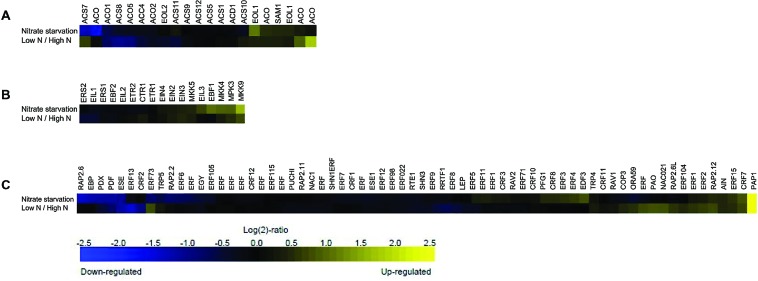
**Meta-profiling hierarchical average of ethylene genes induced in response to different nitrogen (N) perturbations.** The expression profiles of three ethylene gene lists following N conditions were analyzed using the similarity search tool: **(A)** ethylene biosynthesis; **(B)** ethylene signaling; and **(C)** ethylene response. The data consist of the ethylene-related genes represented in the publicly available Affymetrix ATH1 microarrays obtained using the Genevestigator toolbox. Blue and yellow respectively indicate down- and up-regulation; black indicates no change in expression. The values indicate the fold-change in expression in nitrogen starved and untreated rosette samples, and in low- and high-nitrate seedlings.

This meta-profiling showed that the ethylene biosynthetic pathway is regulated by N conditions (**Figure 1A**), and that the genes involved in ethylene biosynthesis appeared to be transcriptionally active under these conditions. In the case of nitrate starvation, ACC synthase *ACS7* and a putative *ACO* (and *ACS10*) were strongly repressed in seedlings, but both were induced in rosette samples treated with low and high N levels, whereas ACC synthases *ACS8* and *ACS4*, and ACC oxidase *ACO1*, *ACO5 and ACO2* were negatively regulated under both conditions. However, *ETO1* (*OVERPRODUCER1*), *SAM1*, *EOL1*-like (ETO-like) and other putative *ACO* were induced in response to N deprivation and low/high N conditions. It is tempting to hypothesize that the multi-gene *ACS* and *ACO* families are both temporally and spatially differentially expressed under low N environmental conditions, as has previously been shown in the case of stresses such as Pi-deprivation (Kim et al., 2008; Roldan et al., 2013), and depend on the species, tissue and developmental stage of the plants (Inaba et al., 2007; Trivellini et al., 2011). A large-scale transcriptome analysis has detected an *ACO6* homolog involved in ethylene synthesis during the early response of cucumber seedlings to N deficiency (Zhao et al., 2015), and the induction of an *ACO4* homolog and *ACO*-like transcript has been observed in response to N starvation in studies of chronic low N conditions (Bi et al., 2007; Peng et al., 2007).

It is also worth noting that suboptimal nutrient supply promotes leaf senescence (Mei and Thimann, 1984; Jibran et al., 2013). Balazadeh et al. (2014) have recently reported that plants undergoing senescence retain the capacity to sense and respond to the availability of N nutrition by reversing the senescence phenotype induced by N starvation. In this study, the expression of *ACS2*, *ACS6*, and *ACS7*, and *ACO2*, *ACO3*, and *ACO4* was increased during senescence, but only *ACS6* was first induced after 4 days of N deficiency and then reduced 3 h after N resupply. *ACO2* and *ACO4* transcript levels were also increased by N deprivation and then significantly down-regulated after 3 days of N resupply, once again highlighting the complexity of ACS and ACO regulation by various stresses signals.

Ethylene production (particularly the rapid breakdown of ACS proteins) is also tightly controlled by means of protein degradation (Christians et al., 2009). The recently characterized *Arabidopsis* mutant *hps3* (Wang et al., 2012), which is hypersensitive to Pi starvation, was previously identified as an allele of the *ETO1* gene that negatively regulates ethylene biosynthesis by producing 10–50 times more ethylene than the wild type (Wang et al., 2004), and our Genevestigator analysis showed that *ETO1* and *EOL1* are weakly expressed under low N conditions. Although these findings potentially define the role of ethylene in regulating multiple plant responses to conditions including N starvation, there is a need for further experimental analyses aimed at identifying the molecular components that interact with ethylene signaling in regulating plant responses to N.

A meta-analyses of the ethylene receptors and mitogen-activated protein kinases (MAPK, MPK, or MKK) linking upstream sensors to the downstream processes of hormonal responses under conditions of N deprivation shows that *ERS2* and *EIL1* are down-regulated, whereas the MPKs involved in ethylene signaling are all induced (**Figure 1B**). Previous studies have shown that MPK3 can be activated by various MKKs that participate in specific signaling pathways: for example the MKK4/MKK5/MKK9 pathway activates MPK3/MPK6 to promote ethylene production (Liu et al., 2008), and MKK9 activates MPK3/MPK6 to regulate leaf senescence (Zhou et al., 2009) and ethylene signaling (Yoo et al., 2008). However, it is not yet known whether MAPK signaling cascades are directly involved in regulating plant responses to various N conditions.

Nitrogen deficiency may play a positive role in ethylene biosynthesis and signaling as *in silico* analysis reveals the slight down-regulation of *CTR1* and up-regulation of *EIN3* under conditions of N starvation and low/high N levels. Zheng et al. (2013) have similarly found that low-level nitrate treatment induces rapid bursts of ethylene production and regulates the expression of the ethylene signaling components *CTR1*, *EIN3* and *EIL1*, and *NRT2*.1 in wild-type plants. The authors used NO_3_^-^ transporter mutants *nrt1.1* and *nrt2.1* and the ethylene mutants *ctr1-1* and *ein3-1eil1-1*, and elegantly proposed that NO_3_^-^ deficiency induces a negative feedback loop between the transcription of *NRT2.1* and ethylene biosynthesis and signaling that allows plants to fine tune nitrate acquisition during the exploration of dynamic soil conditions.

The gene sets specified in **Figure 1C** were further classified into gene ontology (GO) categories in order to help the identification of over-representation. Sixty-nine genes were initially uploaded to the DAVID Bioinformatics Resources 6.7 platform (Huang et al., 2009^2^) in order to identify significantly enriched biological themes by examining enrichment in more than 40 publicly available annotation categories (Trivellini et al., 2012). The analysis identified six clusters that showed significant enrichment, with enrichment scores (ES) ranging from 69.95–1.37 (**Table 1**). The most enriched annotation cluster (ES 69.95) was not surprisingly associated with the genes belong to the Apetala2/ethylene response factor (AP2/ERF-TF) super family; the second cluster contained genes with transcription repressor activity (ES 8.68); the third consisted of genes related the biological processes of root and lateral root development (ES 3.45); the fourth included genes involved in the negative regulation of the ethylene-mediated signaling pathway (ES 2.33); the fifth included genes involved in the response to cytokinin (CK) stimuli (ES 1.49); and the sixth the genes involved in the response to jasmonic acid stimuli (ES 1.39).

**Table 1:** Functional annotation clustering using DAVID bioinformatics resources 6.7.

Cluster number	Enrichment score	Category	Term	Gene count
1	69.95	GOTERM_BP_FAT	Response to ethylene stimulus	61
2	8.68	GOTERM_MF_FAT	Transcription repressor activity	9
3	3.45	GOTERM_BP_FAT	Root and lateral development	6
4	2.33	GOTERM_BP_FAT	Negative regulation of ethylene mediated signaling pathway	3
5	1.49	GOTERM_BP_FAT	Response to cytokinin stimulus	4
6	1.39	GOTERM_BP_FAT	Response to jasmonic acid stimulus	6


*Cluster enrichment scores for selected significantly enriched functional clusters containing terms showing a significant change (*P* < 0.05) after Benjamini–Hochberg correction for multiple testing, and the term(s) most representative of each cluster. The genes associated with the most represented terms are shown in **Supplementary Table S2**.*

The AP2/ERF-TF family is involved in signaling processes and the responses to environmental stresses (Vogel et al., 2012). Most importantly, there is increasing evidence that AP2/ERF proteins are components of multiple signaling pathways as they control the expression of downstream genes and tune cross-talk between the signaling pathways involving macronutrients deficiency (Kim et al., 2012; Cai et al., 2013; Takehisa et al., 2013). Genome-scale transcriptional profiling of cucumber seedlings has shown that seven ERF genes are regulated under conditions of N starvation (Zhao et al., 2015), and comprehensive expression profiling of N starvation-responsive miRNAs has identified miR829.2, which is predicted to target an AP2 domain ethylene response factor required for morphogenesis in the early lateral root primordium of *Arabidopsis* (Liang et al., 2012), thus highlighting the important role of this transcription family in N-starved root development.

Furthermore, as was done in the case of the genes involved in the ethylene biosynthesis and perception machinery, the ethylene response gene list was compared with the publicly available microarray data using the Perturbations tool in the Condition Search toolset of Genevestigator (Hruz et al., 2008). All of the ethylene response genes seemed to be transcriptionally active under condition of N starvation and/or low/high N levels, with the *PURPLE ACID PHOSPHATASE 1 (PAP1)*, *PYRIDOXINE BIOSYNTHESIS 1.1 (PDX1.1)*, *PLANT DEFENSIN 1.2B (PDF1.2B)* and *ETHYLENE AND SALT INDUCIBLE 3 (ESE3)* responding strongly to both types of N perturbation (**Figure 1C**). *PAP1* encodes transcription factors regulating the expression of anthocyanin biosynthetic genes in vegetative tissues. *PAP1* expression is a frequent plant response to stress conditions such as drought, heat, chilling, N deficiency and in response to abscisic acid (ABA), and the sugars in which anthocyanin is accumulated (Luo et al., 2012). The availability of N represses anthocyanin biosynthesis-related gene expression (Rubin et al., 2009) whereas N deficiency stimulates it (Peng et al., 2008). As it is known that ethylene stimulates the expression of the genes related to anthocyanin biosynthesis (Afifi et al., 2003; El-Kereamy et al., 2003), N starvation could induce a transient rise in ethylene production and signaling (Zheng et al., 2013). A new allele of ROOT HAIR DEFECTIVE3 (RHD3) with an anthocyanin over-accumulation phenotype under conditions of N starvation has recently been identified (Wang et al., 2015), and the authors speculate that RHD3 achieves its negative effect on anthocyanin biosynthesis via an ethylene-regulating pathway involving the ETR1, EIN2, and EIN3/EIL1-mediated signaling cascade. Further investigations are needed to clarify the molecular mechanism of RDH3 underlying ethylene signal transduction.

Interestingly, *RAP 2.6*, *RAP 2.3 (EBP)*, and *RAP 2.2* were significantly down-regulated in the N starvation experiment, whereas *RAP2.12* and *RAP2.6L* were weakly up-regulated under both conditions, and *ERF73* seemed to be differentially regulated (**Figure 1C**). These genes of the AP2/ERF family are responsible for modulating tolerance to the hypoxic stresses encountered by plants: *AtRAP2.2* and *RAP2.3* are important for ethylene-mediated tolerance to hypoxia in *Arabidopsis* seedlings (Hinz et al., 2010; Limami et al., 2014), and the same is true of *ERF73*, the hypoxia-responsive *ERF1* gene (*HRE1*) (Licausi et al., 2010); the overexpression of *RAP2.6L* delays the waterlogging induced by premature senescence and may function through the ABI1-mediated ABA signaling pathway (Liu et al., 2012); *RAP2.12* is involved in the activation of hypoxic gene expression and ethylene responses (Licausi et al., 2011; Zhao et al., 2012); and *RAP2.6* is involved in the response to ABA, wounding, jasmonic acid, salt, cold, and osmotic stresses (Fowler and Thomashow, 2002; Zhu et al., 2010). A number of studies have shown that hypoxic stress can be mitigated by nitrate fertilization (Morard et al., 2004; Horchani et al., 2010), but also that nitrate uptake and assimilation can be affected by oxygen-limiting conditions (Oliveira and Sodek, 2013; Oliveira et al., 2013). It therefore seems to be clear that there is a link between N-limiting conditions and the regulation of the genes associated with hypoxia. The ethylene-responsive genes typically involved in hypoxia are potential connectors in the gene/metabolite/hormone-related network of response to N starvation, but further studies are necessary in order to verify their possible role in the N assimilation and signaling pathway.

## Ethylene Responses to Varying Levels of N Availability

The N status of a plant influences its metabolism and growth, and can affect the synthesis of the building block metabolites and the distribution of growth substances. This interdependence is due to the fact that nutrient deficiency or excess affect the concentrations of specific hormones capable of directing the translocation and accumulation of nutrients (Kuiper, 1988; Kuiper et al., 1989), and a similar relationship has also been reported in the case of nutrient and ethylene interactions. Ren et al. (2013) have recently found that the addition of N reduces leaf longevity mainly by altering leaf ethylene production: this result was substantiated by the fact that the cobalt chloride-induced inhibition of ethylene biosynthesis reduced leaf N concentration and leaf longevity, presumably because a high N concentration stimulates the activities of the enzymes associated with ethylene synthesis (Tian et al., 2009). Increased ethylene production may also be involved in modulating nitrate transporters (Tian et al., 2009) and nitrate metabolism (Leblanc et al., 2008) at high nitrate levels. A nitrate concentration of 10 mM increases the expression of genes encoding *ACS* and *ACO* (*AtACS* and *AtACO*), and leads to a sudden increase in ethylene production in *A. thaliana* plants. Furthermore, the upregulation and downregulation of nitrate transporters (AtNRT1.1 and AtNRT2.1) was observed by exogenously applying the ethylene synthesis precursor ACC and AVG in low and high nitrate concentration, respectively, whereas the *etr1-3* and *ein2-1* mutants were insensitive to high nitrate concentrations (Tian et al., 2009). A very interesting study by Misyura et al. (2014) found that ethylene may act as a plant–plant communication signal in rice under conditions of high-density stress, when the expression of ethylene-associated genes was related to ethylene homeostasis. The authors showed that N availability can influence the growth of rice plants dependent on ethylene homeostasis, and that the developmental characteristics of plants were negatively affected under high density conditions when N was limited (3 mM NO_3_^-^) or sufficient (10 mM NO_3_^-^). The availability of N influences the evolution of ethylene and affects photosynthesis, stomatal conductance and growth in *B. juncea* plants (Khan et al., 2008; Iqbal et al., 2011). Field experiments demonstrated that the application of ethephon (ethylene-releasing compound) to plants grown with N levels of 40 and 80 mg N kg^-1^ increased ethylene production and photosynthesis (Iqbal et al., 2011). It has also been suggested that the application of ethephon induces stomatal and carboxylation efficiency and the Calvin cycle enzymes in mustard plants grown at various N levels, with significant interaction between ethylene, N availability, and photosynthetic characteristics.

Improving the acquisition of macronutrients such as N, phosphorus (P) and potassium (K) in poorly fertile soils is one of the main objectives of program aimed at reducing the use of fertilizers and increasing the efficiency of nutrient use (Hirel et al., 2007; Lynch, 2007). The efficient use of N fertilizer is essential to ensure a better return on investment and minimize the adverse effects of accumulated reactive N species on the environment. It is therefore important to increase the N use efficiency (NUE) of plants in order to avoid N wastage and accumulation. NUE, the efficiency of carboxylation and water use, and the dry mass of mustard plants increased at different levels of N in combination with ethephon (Iqbal et al., 2011). Exogenously sourced ethylene enhances photosynthetic NUE and promotes photosynthesis in various types of mustard plant with differing photosynthesising capacity (Iqbal et al., 2012). The application of ethrel at basal 80 kg N ha^-1^ increased the efficiency of N uptake and use in mustard plants, and that the exogenous application of ethephon increased stomatal conductance, photosynthesis and growth under conditions of N deficiency and optimization as a result of increased NUE (Mir, 2002). It has also been found that, under N-deficient conditions, greater endogenous ethylene evolution decreases NUE, photosynthesis and growth in mustard plants (Iqbal et al., 2011). However, high levels of ethylene can also have a negative impact on plant growth and photosynthesis. Under certain conditions the increase of the ethylene sensitivity and ethylene action overcomes N deficiency by increasing photosynthesis and growth in plants with sufficient or deficient N availability (Iqbal et al., 2011). Recently has been reported that N availability regulates ethylene formation, which regulates plant N content and nitrate reductase (NR) activity (Iqbal et al., 2015). Subsequently ethylene increases the proline content and salt tolerance of *B. juncea* plants, and improves photosynthesis and growth.

Nitrogen deficiency leads to strong synergistic interactions between volicitin and ethylene, indicated by the induction of volatile sesquiterpene and indole emissions. Whereas volicitin-induced volatiles are greatly reduced in plants with medium N levels, and there are virtually no interactions with ethylene. The altered volicitin–ethylene interaction due to changes in the magnitude of induced volatile emissions observed in plants with low and medium levels of N availability is consistent with the known increase in ethylene sensitivity that occurs during N deficiency (Schmelz et al., 2003). N deprivation enhances the sensitivity of ethylene-responsive cells in root cortex, thus leading to cell lysis and aerenchyma formation, and that the exogenous application of ethylene (1.0 μL L^-1^) further promoted aerenchyma formation in N-starved roots (He et al., 1992). N starvation increases the number or affinity of root receptors, thus allowing roots to responds to lower concentrations of ethylene than those found in unstressed roots. Plants supplied with high nitrate levels (30 mM) increased their aerial ACC content by translocating it from the roots to the shoot in order to induce ethylene synthesis in the leaves by means of ACC oxidase (Saiz-Fernández et al., 2015). Ethylene plays a role in the regulation of fully developed and expanding leaves by reducing leaf area when ethylene accumulates in developing tissues (Young et al., 2004; He et al., 2009). The interaction between ethylene and N may also increase the synthesis of amino acids, proteins and enzymes. The production of ethylene by soluble solids could be due to increased synthesis of the amino acid cysteine, a precursor of ethylene that may be extended to synthesize other amino acids (Kaack and Pedersen, 2014). Zhao et al. (2015) studied changes in the expression of transcriptional factor and kinase genes at transcriptional level during the early stage of the N deficiency response, and observed seven ERF and three MYB transcription factors, five NAC domain-containing proteins, and four zinc finger proteins. Bi et al. (2007) and Peng et al. (2007) have found that ACO4 and another ACO homologue showed responses to N deficiency: ethylene production generally increases upon N deprivation but, in comparison with explants in standard MS medium, ethylene production by rhizome explants in low N medium was reduced after 1–3 months of culture. Zhang et al. (2013) found low nitrate treatment-induced rapid bursts of ethylene production and the regulated expression of the ethylene signaling components *CTR1,EIN3* and *EIL1* in wild-type *A. thaliana* (Col-0) seedlings, and enhanced ethylene response reporter EBS:GUS activity in Col-0 and the ethylene mutants *ein3-1, eil1-1* and *ctr1-1*. The treatment also caused the up-regulation of *NRT2.1* expression, which was responsible for enhanced high-affinity nitrate uptake, and had a positive effect on ethylene biosynthesis and signaling. However, ethylene down-regulated *NRT2.1* expression and reduced high-affinity nitrate uptake, thus suggesting that nitrate deficiency gives rise to a negative feedback loop between *NRT2.1* expression and ethylene biosynthesis and signaling, which may contribute to the fine tuning of plant nitrate acquisition during the dynamic exploration of soil conditions.

## Ethylene Production in Different Plant Organs at Different Levels of N Availability

Ethylene can be produced in any plant tissue and is modulated by various internal and external factors. The responses of different organs to ethylene vary, depending on tissue sensitivity and the stage of plant development.

## Root Responses

The efficient absorption of macronutrients such as N, and developing the traits involved in remodeling root system architecture in order to acquire N more efficiently, are important targets of modern plant breeding program (Forde, 2014). Phytohormones are involved in controlling root development and architecture by means of N-mediated signals, and recent transcriptomic studies have shown that auxin, ethylene and CK are involved in root architectural responses to nitrates (Tian et al., 2009; Ruffel et al., 2011; Jin et al., 2012). Lemaire et al. (2013) found that ethylene signaling affects nitrate uptake and the expression of *BnNRT* nitrate transporter genes depending on changes in the length of exploratory and root hair systems. Different species, and even the same species under different growing conditions, may have opposite behaviors. In comparison with the wild type, *Never Ripe* (NR) ethylene-insensitive tomato mutants have more below-ground roots and fewer above-ground adventitious roots. Interactions and cross-talk with other plant hormones can lead to different responses. The application of exogenous auxin leads to different behavior (Clark et al., 1999), thus indicating that the effects of ethylene depend on its interaction with auxins as well as abiotic stresses such as nutrient deficiency.

Ethylene deficiency generally induces root development in order to increase the root biomass necessary for exploring a wide area of soil in search of the deficient nutrient. Ethylene can modulate root waving, and the direction and length of root growth (Buer et al., 2003), but the response can be affected by interactions with nutrients. More studies should be carried out in order to investigate root architecture under conditions of N deficiency or excess using ethylene inhibitors. It has been found that N starvation simultaneously increases ethylene evolution and induced aerenchyma formation in the roots of *Zea mays* plants (Drew et al., 2000). Basal roots are more sensitive to ethylene than apical roots (Takahashi et al., 2015). The induction of aerenchyma is also a means of adapting to flooding, and oxygen shortage can initiate programmed cell death (PCD) in roots. Hypoxia associated with N deficiency enhances aerenchyma development, whereas anoxia inhibits or reduces it because the complete lack of oxygen blocks the ACC oxidase enzyme, which catalyzes the last step in ethylene biosynthesis. The use of ethylene biosynthesis and action inhibitors has shown that ethylene is directly involved in PCD in roots (He et al., 1992). High N (especially nitrate) availability in soils induces ethylene biosynthesis in roots. A number of studies have investigated the effects of ethylene and high nitrate content on legumes, in which ethylene biosynthesis inhibits the nodules necessary for N fixation and lateral root development (Caba et al., 1998; Okushima et al., 2011). The effect of the interaction of ethylene and high nitrate concentration on nodule formation has been elegantly demonstrated in various experiments using ethylene activators and biosynthesis inhibitors such as AVG and silver (Peters and Crist-Estes, 1989; Caba et al., 1998; Nukui et al., 2000). The inhibition of nodule formation is negative because it reduces N fixation in leguminous plants; however, from an ecological point of view, plants do not need to develop nodules for gaseous N fixation in soils that are rich in N, particularly nitrates.

Ethylene causes a triple response in *Arabidopsis* roots: the rapid down-regulation of cell elongation, the induction of ectopic root hairs, and an increase in root width. Le et al. (2001) found that slight changes in the concentration of environmental ethylene in *Arabidopsis* modulate the elongation of target cells in root epidermis, and suggested that ethylene is a means of fine and fast tuning root elongation in nature. It has also been demonstrated that C/N balance is involved in root morphogenesis (Malamy and Ryan, 2001; Martin et al., 2002), and that C and N interact with the major plant hormones (Sheen et al., 1999). Le et al. (2001) reported that ethylene inhibits the elongation of root cells, but does not affect root length in the root regions in which cell wall formation occurs before an increase in ethylene level. Increased ethylene synthesis with low concentrations of ACC promotes the initiation of lateral root primordial; however, treatment with higher ACC doses inhibits the formation of new primordia, but promotes the emergence of those already existing (Ivanchenko et al., 2008). N deficiency increases root sensitivity to ethylene and subsequent aerenchyma formation in maize seedlings (He et al., 1992), although ethylene production is reduced (Drew et al., 1989). Tari and Csiszár (2003) have found that, at pH 4.0, nitrite treatment decreases the evolution of ethylene from the root apex but not from the base. Yang et al. (2011) reported that ethylene inhibits NH_4_-stimulated root hair branching, and that ACC 0.04 mM antagonized the effect of NH_4_ by reducing hair branching from the 24% caused by NH_4_NO_3_ to only 5%.

Nitrate can act as both a nutrient and a signal that regulates global gene expression in plant organs. Tian et al. (2009) found that, in the presence of high nitrate levels, roots ethylene production increases from roots as a result of an increase in the expression of the genes encoding *ACS* and *ACO*. They also showed that ethylene regulated nitrate-dependent root development by modulating the expression of nitrate transporters *NRT1.1* and *NRT2.1*, thus demonstrating that ethylene signaling is involved in regulating nitrate uptake on the basis of changes in root elongation. The *etr1-3* and *ein2-1* mutants of ethylene signaling were insensitive to high nitrate concentrations. Lemaire et al. (2013) demonstrated that treatment with the ethylene precursor ACC induces a partial compensatory increase in N uptake, associated with the over-expression of the nitrate transporter genes, *BnNRT2.1* and *BnNRT1.1*. Similar results were obtained by Leblanc et al. (2008) and Le Ny et al. (2013), who suggested that there is a linear correlation between root length and *BnNRT2.1* expression levels in response to 10 μM AVG or changes in nitrate availability. However, Leblanc et al. (2013) reported a decrease in *BnNrt2.1* expression with an increase in ACC concentrations from 0.1 to 10 μM, thus suggesting that *BnNrt2.1* expression may adapt to changes in the absorbing surface of whole mature root by means of a still unknown regulatory mechanism. Leblanc et al. (2008) found that the rapid modulation of root elongation is more dependent on ethylene than on the nitrate signal: ACC treatment reduced C allocation and aspartate content in roots, thus showing that aspartate content correlates with changes in root length and shoot surface area. Canales et al. (2014) reported that up to 10% of the *Arabidopsis* genomes are N responsive, and approximately 7% in maize transcriptomes (Yang et al., 2011). N-induced root developmental plasticity is highly cell specific and finely regulated within the root (Gifford et al., 2008). Among the N responsive gene, five nitrate-responsive genes encoding *NRT2.1*, *NR*, *HB2*, *NiR*, and *HB1* are specifically regulated in the transition zone (Manoli et al., 2014; Trevisan et al., 2014). Trevisan et al. (2015) also reported that the transition zone is critical in sensing nitrate, which directly influences the transcript levels of a few genes and acts indirectly through NR.

Ethylene is also involved in regulating legume–rhizobial interactions: it influences the initial response of root hairs to *Rhizobia* bacteria exposure and the progression of infection into the cortex. Penmetsa and Cook (1997) found that the sickle mutant in *Medicago truncatula* is ethylene-insensitive and hyper-nodulated, and provided genetic support for the involvement of ethylene in regulating rhizobial symbiosis by encoding an ortholog of *EIN2*. Exogenous ethylene severely inhibits the formation and function of N-fixation nodules on legume roots (Peters and Crist-Estes, 1989), possibly because the developmental effects of ethylene include the inhibition of cell division, DNA synthesis, and hook expansion (Apelbaum and Burg, 1972), and the induction of phytoalexin and extension biosynthesis (Ecker and Davis, 1987). Ethylene may act as a secondary signal regulating nodulation on the basis of the N status of the plant and as a negative feedback regulator of rhizobial infection (Oldroyd et al., 2001). Malamy and Ryan (2001) have suggested that the number of lateral roots is reduced in older root regions under conditions of N starvation.

## Leaf Responses

N deficiency also increases ethylene evolution in leaves as a consequence of stress (Legé et al., 1997). Over-irrigation of tomato plants induces N deficiency in leaves and greater ethylene biosynthesis, whereas the use of calcium nitrate to restore adequate N levels reduces ethylene evolution to control levels (Fiebig and Dodd, 2015). N starvation or deficiency also induces leaf senescence (particularly leaf yellowing), promotes the re-mobilization of nutrients from leaves to storage organs, and increases tissue sensitivity to ethylene. Many plants react to N starvation by activating the phenylpropanoid pathway and accumulating anthocyanins (Lea et al., 2007). In particular, low N levels in soils or growing media activates phenylalanine ammonia lyase (PAL, EC 4.3.1.5), the key enzyme of phenylpropanoid compounds. Various post-harvest studies have demonstrated that ethylene stimulates PAL activity, and it is known that the exposure of lettuce to ethylene generates russet spotting (i.e., brown spots on the mid-rib of head lettuce) (Hyodo et al., 1978; Ke and Saltveit, 1988).

There are no specific studies of the effects of the ethylene induced by N deficiency and the consequent PAL activity, but it is reasonable to assume that ethylene plays a pivotal role in this physiological behavior. Studies of *Medicago sativa* cell suspensions treated with 2-aminoindan-2-phosphonic acid (AIP), a potent inhibitor of PAL, have shown enhanced ethylene biosynthesis (Cvikrová et al., 1999), and similar results have been obtained *in vitro* callus cultures of red-fleshed apples with or without N supply in growth media. The absence of N strongly induces anthocyanin accumulation, thus demonstrating the direct role of N on phenylpropanoid activation. Under conditions of N deficiency or starvation, plant leaves activate a N-recycling system in which N is recycled from phenylalanine by means of deamination to cinnamic acid, a reaction that is catalyzed by the PAL enzyme. Although it has not yet been established, it is likely that this futile cycle is under the control of ethylene. Ethylene biosynthesis is also affected by excess N. It is well known that the availability of high N levels induces vegetative growth, makes shoots more susceptible to insect attack (Davies et al., 2004), and causes greater damage due to abiotic stresses. The availability of 20 mM N in *B. juncea* plants increases ACS activity and ethylene evolution (Iqbal et al., 2015).

Plant photosynthesis and sugar biosynthesis have to balance any reduction in nitrates. As uptaken nitrate is reduced to ammonia under conditions of high N levels, plants cannot incorporate all of the reduced ammonia in amino acids, and this can generate stress leading to ethylene biosynthesis.

## Flower Life

There are no specific studies linking N deficiency to ethylene evolution in flowers, but it has been reported that N deficiency increases ethylene biosynthesis and tissue sensitivity. Pre-harvest N deficiency affects the photosynthetic activity of plants, and the life of both growing and cut flowers (Druege, 2000). Under conditions of stress such as N efficiency, plants accelerate all of the physiological processes related to species dispersal: these usually include the induction of flowering in order to ensure dissemination.

No studies of flower senescence under conditions of N deficiency or excess have yet been carried out, but this would be interesting to investigate further.

## Fruit Responses

N availability affects fruit development and quality, in particular low nitrogen content delay ripening and fruits are poor of sugars affecting negatively the overall quality. High nitrogen content usually induces rapid growth and fruits after harvest have faster senescence. Melons harvested from soil with deficient (0 or 50 kg ha^-1^) or excess N (165 kg ha^-1^) levels have the same rate of ethylene biosynthesis. Ethylene production is lowest at the optimal fertilization dose of 110 kg ha^-1^ at harvest, after 8 days of post-harvest storage at 10°C, ethylene production decreased and no significant differences were found among treatments (Ferrante et al., 2008).

It has been found that the use of high, medium or low N fertilization does not affect ethylene production rates in peach trees, but high N levels delay the ripening of the fruit (Okamoto et al., 2001). No differences in ethylene production have been found in apples receiving soil and foliar N, and controls with no supply of N (Wargo et al., 2004). There are few published information concerning N availability and ethylene production in horticultural crops, and so further studies are required in order to establish how N soil content affects ethylene production and the subsequent post-harvest performance of fruit.

**FIGURE 2:**
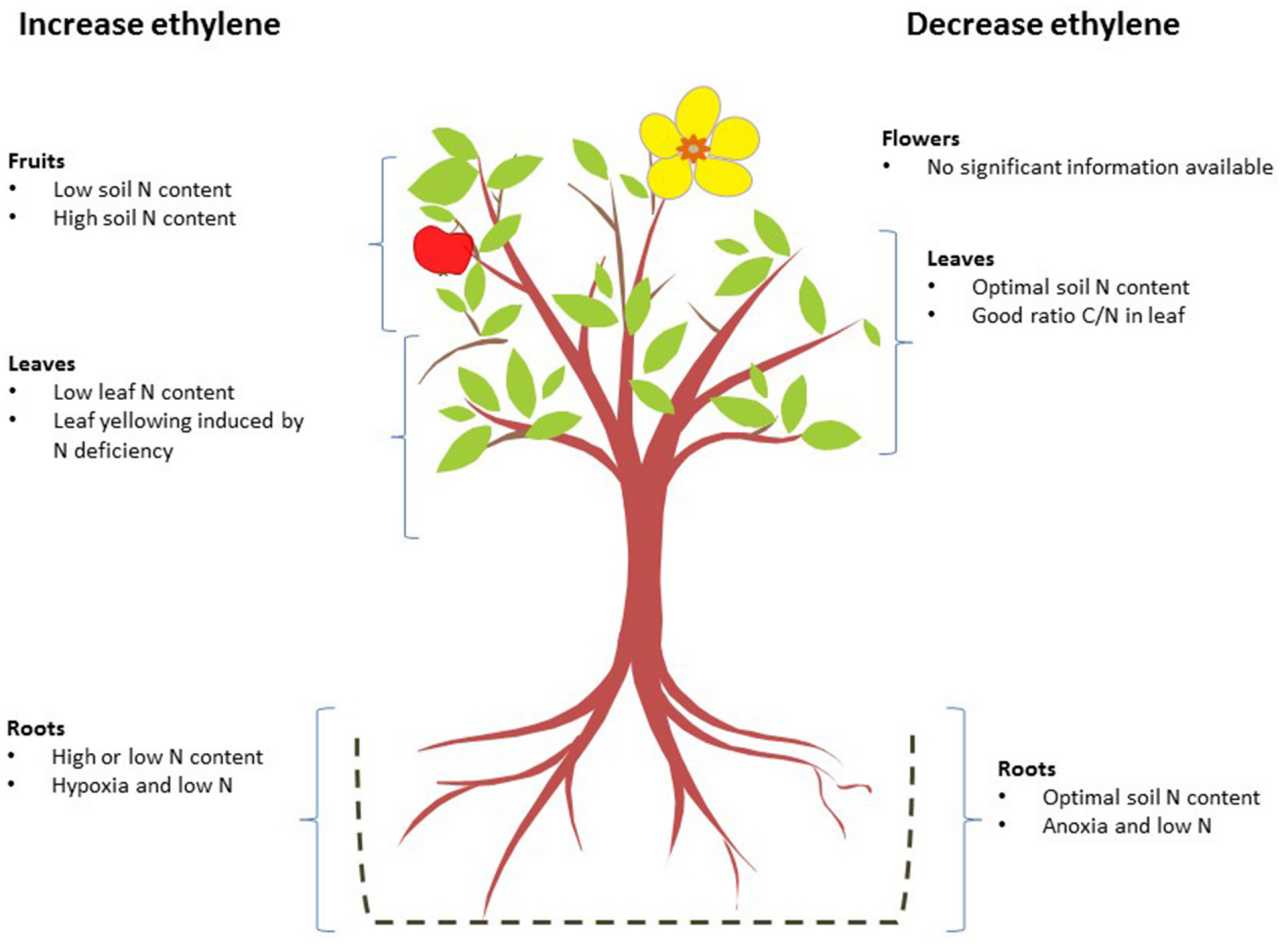
**Effects of nitrogen deficiency and excess on ethylene biosynthesis in different organs.** The nitrogen levels that, alone or in combination with other factors, increase (left) or reduce ethylene biosynthesis (right) are shown for each organ.

**Figure 2** shows a general view of how ethylene concentrations are affected by N levels in the different parts of plants.

## Ethylene Interactions with Other Phytohormones at Different Levels of N Availability

Nitrogen levels considerably influence root architecture and crop production (Mohd-Radzman et al., 2013), and plants have efficient internal execution points to control N uptake, reduction and assimilation, and environmental NUE (Xu et al., 2012). Some recently published studies have begun to elucidate the link between ethylene signaling and N availability. Nitrate assimilation can take place directly in roots, and the assimilated nitrate can be stored in vacuoles or transferred to the aerial parts of a plant (Krapp, 2015) but, in many species, it preferentially occurs in shoots, where photosynthesis takes place and energy is easily available (Searles and Bloom, 2003).

However, a number of stresses can separate nitrate assimilation and photosynthesis by triggering nitrate allocation to roots. As hormonal action is an interdependent process, the action of ethylene at different N levels may be influenced by regulatory interactions between ethylene and other phytohormones. Genetic studies of *A. thaliana* by Swarup et al. (2007) have shown that ethylene-induced inhibition of root growth involves auxin, the presence of which significantly enhanced the inhibition of root cell elongation induced by the ethylene precursor ACC. It has also been reported the mutations in auxin transport or signaling components cause aberrant responses to ethylene, thus indicating the existence of cross-talk between the two phytohormones (Luschnig et al., 1998; Stepanova et al., 2005). Alonso et al. (2003) found that mutations in the auxin receptor TIR1 lead to ethylene-insensitive root growth phenotypes. Ethylene inhibits cell elongation by locally stimulating auxin biosynthesis and basipetal auxin transport toward the elongation zone; in mutants deficient in auxin perception or basipetal auxin transport, ethylene cannot activate the auxin response or regulate root growth (Růžička et al., 2007).

Stress-initiated nitrate allocation to roots (SINAR) improves stress tolerance and decreases plant growth under non-stressed conditions via an *ET/JA-NRT1.5/NRT1.8* signaling module (Zhang et al., 2014), which allows the regulation of nitrate assimilation at the level of the organ at which stresses initiate ET and JA signaling, which converges to *EIN3/EIN3-Like1 (EIL1)* in order to modulate *ERF* expression and up-regulate *NRT1.8*; ET and JA signaling mediates the down-regulation of *NRT1.5* via *EIN3/EIL1* and other unknown component(s). Krouk et al. (2010) demonstrated that, at low nitrate levels, mutations in the *NRT1.1* nitrate transporter enhance auxin accumulation in lateral roots and lateral root growth. At low (but not high) nitrate levels, *NRT1.1* represses lateral root growth by promoting the outward transport of basipetal auxin. Ma et al. (2014) showed that low N induces lateral root growth in *Arabidopsis*, and that this growth was dependent on the function of the auxin biosynthesis gene tryptophan aminotransferase related 2 (*TAR2*), which is induced under low N conditions. It has also been shown that molecules mediating auxin influx (AUX1, LAX2, LAX3) and eﬄux (PIN1, PIN2, PIN4, and PIN7) are transcriptionally regulated by N and/or C (Gutierrez et al., 2007; Li et al., 2011), and that most of these carriers are required for root development (Petrášek and Friml, 2009). It has been reported that jasmonic acid is a negative regulator of nodulation. Mi et al. (2008) reported that, in the presence of low N levels, the auxin, CK and nitric oxide (NO) signaling pathways are involved in regulating root elongation: an abundant N supply increases CK levels, but decreases auxin and NO levels in the roots of maize. The exogenous supply of CK increases ethylene production (Stenlid, 1982; Bertell et al., 1990). Nitrate-induced inhibition of root elongation in maize is significantly reversed by treating the roots with a NO donor (SNP) and IAA (Zhao et al., 2007). In the presence of high nitrate levels, endogenous levels of NO in the root apices of maize seedlings are much lower than those in apices grown in the presence of low nitrate levels. The inhibition of NO synthesis reduces root elongation in maize plants grown in a low-nitrate medium (Mi et al., 2008).

It has been reported that N supplementation induces CK accumulation in detached *Helianthus annuus* and *Nicotiana tabacum* leaves (Salama and Wareing, 1979; Singh et al., 1992). *AtIPT3* (a gene involved in CK biosynthesis) is nitrate inducible, and *atipt3* mutants reduce CK levels, thus indicating that *AtIPT3* is a key determinant of nitrate-dependent CK biosynthesis (Miyawaki et al., 2004; Takei et al., 2004). The nitrate transporter *NRT1.1* mediates the nitrate inducible expression of *AtIPT3* (Liu et al., 1999; Ho et al., 2009), and Kiba et al. (2011) have found that CK represses the nitrate transporter gene and nitrate uptake regardless of plant N status. However, Rubio et al. (2009) and Krouk et al. (2011) have shown that CK also regulates the expression of N uptake- and assimilation-related genes, as well as root architecture (Werner et al., 2003; Higuchi et al., 2004). CK may function as a long-distance “root-to-shoot” signal related to NO_3_^-^ supply (Takei et al., 2004; Sakakibara, 2006), and Ruffel et al. (2011) found that it is a crucial component of a root-shoot-root signaling mechanism that is involved in conveying a plant’s NO_3_^-^ status, thus enabling a compensatory increase in lateral root growth in NO_3_^-^-rich zones of a root system foraging for N resources in a heterogeneous N environment.

Signora et al. (2001) showed that both ABA-insensitive mutants (*abi4-1*, *abi4-2*, and *abi5-1*) and ABA-deficient mutants (*aba1-1*, *aba2-3*, *aba2-4*, and *aba3-2*) are less sensitive to the inhibitory effects of high nitrate levels, and the study of the *Medicago truncatula latd* mutant by Yendrek et al. (2010) provided another line of evidence supporting a link between ABA and N signaling. The *latd* mutant is characterized by severe defects in root meristem maintenance and root growth, and its primary root growth is insensitive to nitrate; the *LATD* gene encodes a transporter belonging to the NRT1 (PTR) family, and is rescued by exogenous ABA (Bright et al., 2005; Liang et al., 2007).

Sun et al. (2015) have found that the NR pathway in *Oryza sativa* generates NO, which improves N acquisition capacity by increasing the initiation of lateral roots and the uptake of inorganic N, a strategy that allows the plants to adapt to a fluctuating nitrate supply and increase NUE. Liu et al. (2010) reported that ethylene induces NO formation in *A. thaliana*. N availability affects ethylene biosynthesis and signaling, which further increases N uptake and transport to enhance plant growth. The effect of ethylene on root architecture increases N absorption and influences N transport-related genes. It has been shown that the action of ethylene on N uptake or root growth is not independent of other phytohormones as low nitrate levels also increase CK, auxin, ABA and NO. CK increases the production of ethylene, which acts in coordination with auxin in order to ensure root growth and lateral root formation. These hormones are influenced by N and affect root growth but, as their cross-talk allows them to acquire N in the case of a limited supply, there is a need to verify whether ethylene functions in coordination with these hormones under the same conditions. Inhibiting these phytohormones under condition of limited N availability could provide insights into their mechanism of action, and enable the use of ethylene as a means of increasing plant NUE, avoiding N wastage, and preventing environmental pollution.

It is widely recognized that a high ethylene concentration is a potent inhibitor of nodule development in plants (Lee and LaRue, 1992; Sun et al., 2006; Gresshoff et al., 2009). Ferguson et al. (2011) have recently reported that there is a close relationship between gibberellin, ethylene and nodulation in *Pisum sativum*: the application of the ethylene precursor ACC significantly reduces the number of nodules and root and shoot length in wild-type NA plants, whereas treatment with the ethylene biosynthesis inhibitor AVG increases the number of nodules to 36 times the number formed on gibberellin-deficient mutant *na-1* plants. They also suggested that ethylene biosynthesis genes (*PsACS1* and *PsACO1*) were decreased in *na-1* roots, but the fact that there was no significant change in *PsACO1* in the roots of *na-1* plants during nodule formation indicates that ethylene plays a role in nodulation. However, there is a need for further experiments aimed at investigations the relationships between ethylene and other hormones in nodule formation.

The use of methyl jasmonate restrains nodulation in *Lotus japonicas*, including infection thread formation and *NIN* gene expression in wild-type plants and the hyper-nodulated *har1* mutant (Nakagawa and Kawaguchi, 2006). It has also been found that nodulation is inhibited in *Medicago truncatula* cultured in a growth medium containing JA, and that the number of *NIN* transcripts is larger in transgenic than in wild-type plants. Ethylene signaling negatively regulates the early stage of nodule development, including infection thread formation and the emergence of nodule primordia (Penmetsa and Cook, 1997).

## Conclusion and Future Prospects

Nitrogen availability has a strong influence on ethylene biosynthesis and signaling, and plants have different metabolic responses to optimal and stressful conditions. As N is a core mineral nutrient, larger amounts of N improve crop productivity, increasing NUE. Modulating ethylene availability should have a positive effect on sustainable development by reducing the wastage of applied N and increasing environmental protection. In order to maximize the genetic potential of N use, it is important to focus research on clarifying the interactions of N, ethylene and other hormones in order to be able to use more N under N-deficient conditions without affecting environmental safety.

## Conflict of Interest Statement

The authors declare that the research was conducted in the absence of any commercial or financial relationships that could be construed as a potential conflict of interest.

## Footnotes

1 https://www.arabidopsis.org/

2 http://david.abcc.ncifcrf.gov/
